# Crystal structure of 3-(3,4-di­methyl­anilino)-2-benzo­furan-1(3*H*)-one

**DOI:** 10.1107/S2056989015009299

**Published:** 2015-05-20

**Authors:** Muhammad Salim, Muhammad Nawaz Tahir, Muhammad Shahid, Munawar Ali Munawar

**Affiliations:** a Department of Chemistry, University of the Punjab, Lahore, Punjab, Pakistan; b Department of Physics, University of Sargodha, Sargodha, Punjab, Pakistan

**Keywords:** crystal structure, 2-benzo­furan­one, hydrogen bonding

## Abstract

In the title compound, C_16_H_15_NO_2_, the 2-benzo­furan-1(3*H*)-one and 3,4-di­methyl­aniline fragments are oriented with a dihedral angle of 89.12 (5)°. N—H⋯O hydrogen-bond inter­actions join mol­ecules into *C*(6) chains propagating along the *a* axis. In addition, there are π–π stacking inter­actions between the 2-benzo­furan­one benzene rings [centroid–centroid dis­tance = 3.7870 (13) Å] and C—H⋯π inter­actions between one of the methyl groups and the 3,4-di­methyl­aniline benzene ring.

## Related literature   

For related crystal structures, see: Li *et al.* (2009 ▸); Odabaşoğlu & Büyükgüngör (2006*a* ▸,*b* ▸, 2007*a* ▸,*b* ▸). For graph-set notation, see: Bernstein *et al.* (1995 ▸).
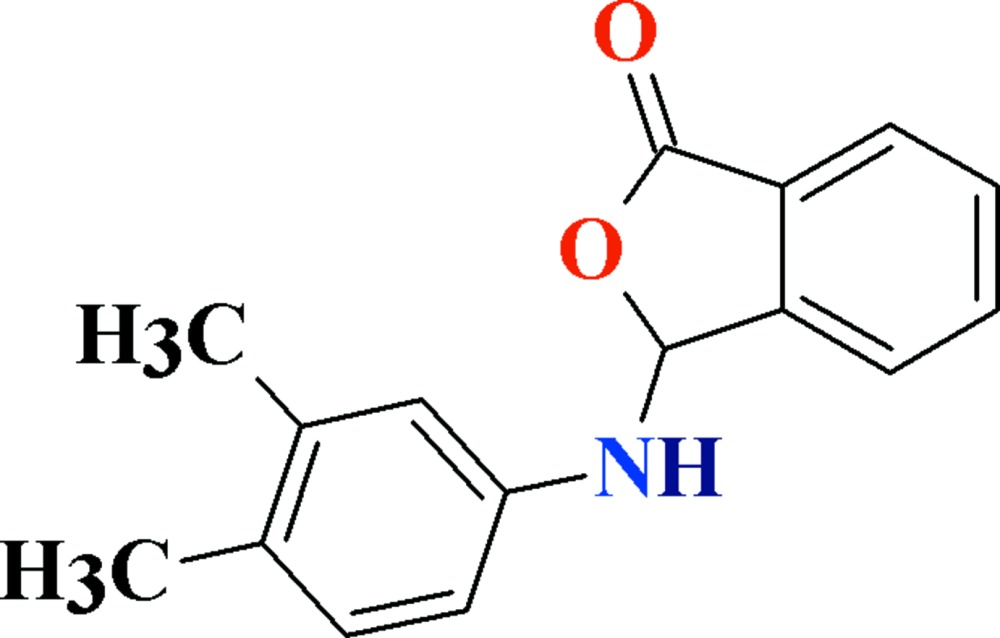


## Experimental   

### Crystal data  

C_16_H_15_NO_2_
*M* *_r_* = 253.29
Orthorhombic, 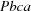

*a* = 7.3386 (7) Å
*b* = 14.9133 (11) Å
*c* = 24.3322 (18) Å
*V* = 2663.0 (4) Å^3^
*Z* = 8
Mo *K*α radiation
μ = 0.08 mm^−1^
*T* = 296 K
0.38 × 0.23 × 0.16 mm

### Data collection  

Bruker Kappa APEXII CCD diffractometer
Absorption correction: multi-scan (*SADABS*; Bruker, 2005 ▸) *T*_min_ = 0.970, *T*_max_ = 0.988
20839 measured reflections
2906 independent reflections
1660 reflections with *I* > 2σ(*I*)
*R*_int_ = 0.042

### Refinement  

*R*[*F* ^2^ > 2σ(*F* ^2^)] = 0.048
*wR*(*F* ^2^) = 0.144
*S* = 1.02
2906 reflections
174 parameters
H-atom parameters constrained
Δρ_max_ = 0.27 e Å^−3^
Δρ_min_ = −0.25 e Å^−3^

### 

Data collection: *APEX2* (Bruker, 2007 ▸); cell refinement: *SAINT* (Bruker, 2007 ▸); data reduction: *SAINT*; program(s) used to solve structure: *SHELXS97* (Sheldrick, 2008 ▸); program(s) used to refine structure: *SHELXL2014* (Sheldrick, 2015 ▸); molecular graphics: *ORTEP-3 for Windows* (Farrugia, 2012 ▸) and *PLATON* (Spek, 2009 ▸); software used to prepare material for publication: *WinGX* (Farrugia, 2012 ▸) and *PLATON*.

## Supplementary Material

Crystal structure: contains datablock(s) global, I. DOI: 10.1107/S2056989015009299/gk2634sup1.cif

Structure factors: contains datablock(s) I. DOI: 10.1107/S2056989015009299/gk2634Isup2.hkl

Supporting information file. DOI: 10.1107/S2056989015009299/gk2634Isup3.cml

**Fig. 1.**
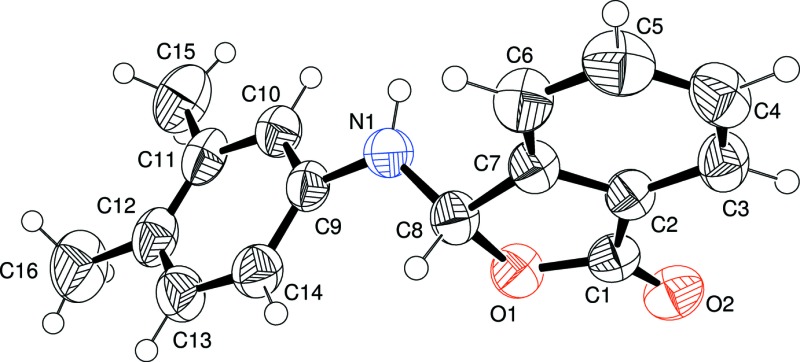
Molecular structure with the atom numbering scheme. The displacement ellipsoids are drawn at the 50% probability level. H-atoms are shown by small circles of arbitrary radii.

**Fig. 2.**
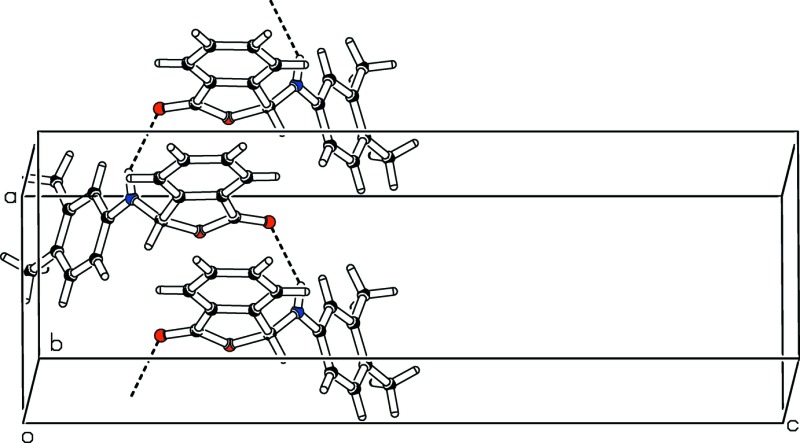
The chains of molecules along the a axis via N—H···O hydrogen bond (PLATON; Spek, 2009).

. DOI: 10.1107/S2056989015009299/gk2634fig1.tif
Mol­ecular structure with the atom numbering scheme. The displacement ellipsoids are drawn at the 50% probability level. H-atoms are shown by small circles of arbitrary radii.

a via PLATON . DOI: 10.1107/S2056989015009299/gk2634fig2.tif
The chains of mol­ecules along the *a* axis *via* N—H⋯O hydrogen bond (*PLATON*; Spek, 2009).

CCDC reference: 1401225

Additional supporting information:  crystallographic information; 3D view; checkCIF report

## Figures and Tables

**Table 1:** Hydrogen-bond geometry (, ) *Cg*3 is the centroid of the C9C14

*D*H*A*	*D*H	H*A*	*D* *A*	*D*H*A*
N1H1O2^i^	0.86	2.31	3.025(2)	141
C15H15*B* *Cg*3^ii^	0.96	2.88	3.661(3)	139

Symmetry codes: (i) 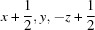; (ii) 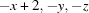.

## supplementary crystallographic information

### S1. Comment

The crystal structures of 3-anilinoisobenzofuran-1(3*H*)-one (Odabaşoğlu & Büyükgüngör, 2006*a*), 3-(2,6-dimethylanilino)isobenzofuran-1(3*H*) -one (Odabaşoğlu & Büyükgüngör, 2006*b*), 3-(4-methylanilino)isobenzo furan-1(3*H*)-one (Odabaşoğlu & Büyükgüngör, 2007*a*), 3-(2- (hydroxymethyl)anilino)isobenzofuran-1(3*H*)-one (Odabaşoğlu & Büyükgüngör, 2007*b*), and 3-((3-oxo-1,3-dihydroisobenzofuran-1-yl)amino) benzoic acid Li *et al.*, 2009) have been published which are related to the title compound (I, Fig. 1). The title compound was synthesized for the biological studies and for the preparation of further derivitives.

The benzofuran ring A (C1–C8/O1/O2) and the 3,4-dmethylaniline group B (C9–C16/N1) are planar with r. m. s. deviation of 0.0209 and 0.0101 Å, respectively. The dihedral angle between A/B fragments is 89.12 (5)°. Intermolecular hydrogen bond of N—H···O type generates *C* (6) chains (Bernstein *et al.*, 1995) along the crystallographic *a* axis (Table 1, Fig. 2). The π–π interactions are observed between the 2-benzofuranone fragments [ *Cg*1—*Cg*2^i^ 3.6204 (12) Å; *Cg*1—*Cg*1^i^ 3.8138 (13) Å; *Cg*2—*Cg*2^i^ 3.7870 (13) Å, *Cg*1 - centroid of C1/C2/C7/C8/O1, *Cg*2 - centroid of C2–C7); i = - 1/2 + *x*, *y*, 1/2 - *z* ]. In addition there are also C—H···π interactions (Table 1).

### S2. Experimental

Equimolar quantities of 3,4-dimethylaniline (0.605 g. 5 mmol) and 2-formylbenzoic acid (0.751 g, 5 mmol) were stirred in methanol for 2 h. The solution was kept at room temperature for crystallization which afforded light brown needles after 48 h.

### S3. Refinement

The H atoms were positioned geometrically (C–H = 0.93–0.96 Å, N—H= 0.86 Å) and refined as riding on their carriers with *U*_iso_(H) = *xU*_eq_(C, N), where *x* = 1.5 for methyl and *x* =1.2 for other H-atoms.

### Crystal data

**Table:**

C_16_H_15_NO_2_	*D*_x_ = 1.264 Mg m^−^^3^
*M**_r_* = 253.29	Mo *K*α radiation, λ = 0.71073 Å
Orthorhombic, *P**b**c**a*	Cell parameters from 4689 reflections
*a* = 7.3386 (7) Å	θ = 1.4–27.0°
*b* = 14.9133 (11) Å	µ = 0.08 mm^−^^1^
*c* = 24.3322 (18) Å	*T* = 296 K
*V* = 2663.0 (4) Å^3^	Needle, light brown
*Z* = 8	0.38 × 0.23 × 0.16 mm
*F*(000) = 1072	

### Data collection

**Table:**

Bruker Kappa APEXII CCD diffractometer	2906 independent reflections
Radiation source: fine-focus sealed tube	1660 reflections with *I* > 2σ(*I*)
Graphite monochromator	*R*_int_ = 0.042
Detector resolution: 7.80 pixels mm^-1^	θ_max_ = 27.0°, θ_min_ = 2.7°
ω scans	*h* = −5→9
Absorption correction: multi-scan (*SADABS*; Bruker, 2005)	*k* = −19→18
*T*_min_ = 0.970, *T*_max_ = 0.988	*l* = −31→31
20839 measured reflections	

### Refinement

**Table:**

Refinement on *F*^2^	Primary atom site location: structure-invariant direct methods
Least-squares matrix: full	Secondary atom site location: difference Fourier map
*R*[*F*^2^ > 2σ(*F*^2^)] = 0.048	Hydrogen site location: inferred from neighbouring sites
*wR*(*F*^2^) = 0.144	H-atom parameters constrained
*S* = 1.02	*w* = 1/[σ^2^(*F*_o_^2^) + (0.0675*P*)^2^ + 0.3907*P*] where *P* = (*F*_o_^2^ + 2*F*_c_^2^)/3
2906 reflections	(Δ/σ)_max_ < 0.001
174 parameters	Δρ_max_ = 0.27 e Å^−^^3^
0 restraints	Δρ_min_ = −0.25 e Å^−^^3^

### Special details

**Table:**

Geometry. All e.s.d.'s (except the e.s.d. in the dihedral angle between two l.s. planes) are estimated using the full covariance matrix. The cell e.s.d.'s are taken into account individually in the estimation of e.s.d.'s in distances, angles and torsion angles; correlations between e.s.d.'s in cell parameters are only used when they are defined by crystal symmetry. An approximate (isotropic) treatment of cell e.s.d.'s is used for estimating e.s.d.'s involving l.s. planes.
Refinement. Refinement of *F*^2^ against ALL reflections. The weighted *R*-factor *wR* and goodness of fit *S* are based on *F*^2^, conventional *R*-factors *R* are based on *F*, with *F* set to zero for negative *F*^2^. The threshold expression of *F*^2^ > σ(*F*^2^) is used only for calculating *R*-factors(gt) *etc*. and is not relevant to the choice of reflections for refinement. *R*-factors based on *F*^2^ are statistically about twice as large as those based on *F*, and *R*-factors based on ALL data will be even larger.

### Fractional atomic coordinates and isotropic or equivalent isotropic displacement parameters (Å^2^)

**Table:**

	*x*	*y*	*z*	*U*_iso_*/*U*_eq_	
O1	0.80336 (19)	0.14473 (8)	0.23068 (5)	0.0528 (4)	
O2	0.8396 (2)	0.16231 (9)	0.32129 (5)	0.0614 (4)	
N1	0.9466 (2)	0.15132 (10)	0.14175 (6)	0.0533 (5)	
H1	1.0572	0.1688	0.1371	0.064*	
C1	0.8541 (3)	0.19202 (12)	0.27513 (7)	0.0457 (5)	
C2	0.9241 (3)	0.27938 (11)	0.25746 (7)	0.0424 (5)	
C3	0.9848 (3)	0.35141 (12)	0.28845 (8)	0.0523 (5)	
H3	0.9871	0.3488	0.3266	0.063*	
C4	1.0416 (3)	0.42698 (13)	0.26083 (9)	0.0610 (6)	
H4	1.0827	0.4765	0.2805	0.073*	
C5	1.0381 (3)	0.42990 (13)	0.20393 (10)	0.0651 (6)	
H5	1.0775	0.4815	0.1860	0.078*	
C6	0.9774 (3)	0.35782 (12)	0.17306 (8)	0.0575 (6)	
H6	0.9761	0.3602	0.1349	0.069*	
C7	0.9189 (3)	0.28247 (11)	0.20076 (7)	0.0443 (5)	
C8	0.8370 (3)	0.19769 (12)	0.17912 (7)	0.0472 (5)	
H8	0.7199	0.2115	0.1617	0.057*	
C9	0.8834 (3)	0.07697 (11)	0.11132 (7)	0.0463 (5)	
C10	1.0086 (3)	0.01250 (12)	0.09499 (7)	0.0521 (5)	
H10	1.1299	0.0189	0.1054	0.062*	
C11	0.9584 (4)	−0.06119 (12)	0.06359 (7)	0.0572 (6)	
C12	0.7769 (4)	−0.07110 (13)	0.04754 (7)	0.0618 (6)	
C13	0.6529 (3)	−0.00635 (14)	0.06404 (8)	0.0632 (6)	
H13	0.5317	−0.0124	0.0535	0.076*	
C14	0.7027 (3)	0.06721 (13)	0.09569 (7)	0.0553 (6)	
H14	0.6161	0.1093	0.1063	0.066*	
C15	1.1015 (4)	−0.12910 (15)	0.04791 (10)	0.0846 (8)	
H15A	1.2147	−0.1140	0.0654	0.127*	
H15B	1.1175	−0.1289	0.0087	0.127*	
H15C	1.0635	−0.1877	0.0596	0.127*	
C16	0.7133 (4)	−0.15104 (15)	0.01424 (10)	0.0953 (9)	
H16A	0.5847	−0.1462	0.0076	0.143*	
H16B	0.7378	−0.2052	0.0342	0.143*	
H16C	0.7769	−0.1524	−0.0202	0.143*	

### Atomic displacement parameters (Å^2^)

**Table:**

	*U*^11^	*U*^22^	*U*^33^	*U*^12^	*U*^13^	*U*^23^
O1	0.0623 (10)	0.0370 (7)	0.0592 (8)	−0.0047 (6)	0.0027 (7)	−0.0033 (6)
O2	0.0646 (11)	0.0614 (9)	0.0581 (8)	0.0045 (7)	0.0074 (7)	0.0143 (7)
N1	0.0515 (11)	0.0518 (9)	0.0566 (9)	−0.0076 (8)	0.0071 (8)	−0.0179 (7)
C1	0.0457 (12)	0.0391 (10)	0.0524 (11)	0.0075 (9)	0.0045 (9)	0.0000 (9)
C2	0.0434 (12)	0.0356 (9)	0.0481 (10)	0.0067 (9)	−0.0002 (9)	−0.0037 (8)
C3	0.0577 (14)	0.0450 (11)	0.0542 (10)	0.0084 (10)	−0.0029 (10)	−0.0122 (9)
C4	0.0638 (16)	0.0385 (11)	0.0809 (15)	−0.0005 (10)	−0.0047 (12)	−0.0147 (10)
C5	0.0725 (17)	0.0377 (11)	0.0851 (15)	−0.0050 (11)	0.0028 (13)	0.0061 (10)
C6	0.0714 (16)	0.0458 (11)	0.0552 (11)	−0.0007 (11)	0.0001 (11)	0.0060 (9)
C7	0.0481 (12)	0.0355 (9)	0.0493 (10)	0.0038 (9)	−0.0016 (9)	−0.0025 (8)
C8	0.0524 (13)	0.0405 (9)	0.0487 (10)	0.0021 (9)	−0.0026 (9)	−0.0040 (8)
C9	0.0561 (14)	0.0444 (10)	0.0385 (9)	−0.0047 (10)	0.0014 (9)	−0.0042 (8)
C10	0.0591 (14)	0.0495 (11)	0.0476 (10)	0.0010 (11)	0.0013 (10)	−0.0049 (9)
C11	0.0845 (18)	0.0456 (11)	0.0417 (10)	0.0002 (12)	0.0104 (11)	−0.0029 (8)
C12	0.0906 (19)	0.0489 (12)	0.0460 (10)	−0.0145 (13)	0.0064 (12)	−0.0084 (9)
C13	0.0706 (17)	0.0646 (13)	0.0543 (11)	−0.0185 (13)	−0.0032 (11)	−0.0076 (10)
C14	0.0604 (15)	0.0512 (11)	0.0542 (11)	−0.0034 (11)	0.0041 (10)	−0.0084 (9)
C15	0.119 (2)	0.0601 (13)	0.0749 (14)	0.0175 (14)	0.0172 (16)	−0.0143 (12)
C16	0.135 (3)	0.0685 (15)	0.0821 (15)	−0.0301 (16)	0.0022 (17)	−0.0282 (13)

### Geometric parameters (Å, º)

**Table:**

O1—C1	1.344 (2)	C8—H8	0.9800
O1—C8	1.503 (2)	C9—C14	1.387 (3)
O2—C1	1.212 (2)	C9—C10	1.388 (3)
N1—C8	1.397 (2)	C10—C11	1.388 (3)
N1—C9	1.412 (2)	C10—H10	0.9300
N1—H1	0.8600	C11—C12	1.395 (3)
C1—C2	1.465 (3)	C11—C15	1.509 (3)
C2—C7	1.381 (2)	C12—C13	1.386 (3)
C2—C3	1.386 (2)	C12—C16	1.515 (3)
C3—C4	1.377 (3)	C13—C14	1.389 (3)
C3—H3	0.9300	C13—H13	0.9300
C4—C5	1.385 (3)	C14—H14	0.9300
C4—H4	0.9300	C15—H15A	0.9600
C5—C6	1.385 (3)	C15—H15B	0.9600
C5—H5	0.9300	C15—H15C	0.9600
C6—C7	1.379 (2)	C16—H16A	0.9600
C6—H6	0.9300	C16—H16B	0.9600
C7—C8	1.496 (2)	C16—H16C	0.9600
			
C1—O1—C8	110.53 (13)	C14—C9—C10	118.79 (17)
C8—N1—C9	122.76 (17)	C14—C9—N1	122.70 (17)
C8—N1—H1	118.6	C10—C9—N1	118.48 (19)
C9—N1—H1	118.6	C9—C10—C11	122.0 (2)
O2—C1—O1	121.98 (17)	C9—C10—H10	119.0
O2—C1—C2	128.89 (17)	C11—C10—H10	119.0
O1—C1—C2	109.12 (15)	C10—C11—C12	119.4 (2)
C7—C2—C3	121.78 (16)	C10—C11—C15	119.1 (2)
C7—C2—C1	108.25 (15)	C12—C11—C15	121.5 (2)
C3—C2—C1	129.96 (17)	C13—C12—C11	118.15 (18)
C4—C3—C2	117.78 (18)	C13—C12—C16	120.0 (2)
C4—C3—H3	121.1	C11—C12—C16	121.8 (2)
C2—C3—H3	121.1	C12—C13—C14	122.5 (2)
C3—C4—C5	120.53 (18)	C12—C13—H13	118.7
C3—C4—H4	119.7	C14—C13—H13	118.7
C5—C4—H4	119.7	C9—C14—C13	119.1 (2)
C6—C5—C4	121.57 (19)	C9—C14—H14	120.4
C6—C5—H5	119.2	C13—C14—H14	120.4
C4—C5—H5	119.2	C11—C15—H15A	109.5
C7—C6—C5	117.87 (18)	C11—C15—H15B	109.5
C7—C6—H6	121.1	H15A—C15—H15B	109.5
C5—C6—H6	121.1	C11—C15—H15C	109.5
C6—C7—C2	120.46 (16)	H15A—C15—H15C	109.5
C6—C7—C8	129.93 (16)	H15B—C15—H15C	109.5
C2—C7—C8	109.55 (15)	C12—C16—H16A	109.5
N1—C8—C7	114.58 (17)	C12—C16—H16B	109.5
N1—C8—O1	112.19 (14)	H16A—C16—H16B	109.5
C7—C8—O1	102.49 (13)	C12—C16—H16C	109.5
N1—C8—H8	109.1	H16A—C16—H16C	109.5
C7—C8—H8	109.1	H16B—C16—H16C	109.5
O1—C8—H8	109.1		
			
C8—O1—C1—O2	−179.85 (17)	C2—C7—C8—N1	−123.94 (17)
C8—O1—C1—C2	0.2 (2)	C6—C7—C8—O1	−179.40 (19)
O2—C1—C2—C7	178.4 (2)	C2—C7—C8—O1	−2.2 (2)
O1—C1—C2—C7	−1.6 (2)	C1—O1—C8—N1	124.55 (17)
O2—C1—C2—C3	−2.8 (4)	C1—O1—C8—C7	1.1 (2)
O1—C1—C2—C3	177.13 (18)	C8—N1—C9—C14	30.1 (3)
C7—C2—C3—C4	−0.4 (3)	C8—N1—C9—C10	−151.93 (17)
C1—C2—C3—C4	−179.03 (19)	C14—C9—C10—C11	0.0 (3)
C2—C3—C4—C5	−0.2 (3)	N1—C9—C10—C11	−178.05 (16)
C3—C4—C5—C6	0.2 (3)	C9—C10—C11—C12	0.3 (3)
C4—C5—C6—C7	0.4 (3)	C9—C10—C11—C15	−179.31 (18)
C5—C6—C7—C2	−1.0 (3)	C10—C11—C12—C13	−0.3 (3)
C5—C6—C7—C8	176.0 (2)	C15—C11—C12—C13	179.33 (18)
C3—C2—C7—C6	1.0 (3)	C10—C11—C12—C16	−178.75 (18)
C1—C2—C7—C6	179.92 (18)	C15—C11—C12—C16	0.9 (3)
C3—C2—C7—C8	−176.51 (17)	C11—C12—C13—C14	0.0 (3)
C1—C2—C7—C8	2.4 (2)	C16—C12—C13—C14	178.46 (19)
C9—N1—C8—C7	−171.10 (16)	C10—C9—C14—C13	−0.3 (3)
C9—N1—C8—O1	72.6 (2)	N1—C9—C14—C13	177.65 (16)
C6—C7—C8—N1	58.8 (3)	C12—C13—C14—C9	0.3 (3)

### Hydrogen-bond geometry (Å, º)

Cg3 is the centroid of the C9—C14

**Table:**

*D*—H···*A*	*D*—H	H···*A*	*D*···*A*	*D*—H···*A*
N1—H1···O2^i^	0.86	2.31	3.025 (2)	141
C15—H15*B*···*Cg*3^ii^	0.96	2.88	3.661 (3)	139

Symmetry codes: (i) *x*+1/2, *y*, −*z*+1/2; (ii) −*x*+2, −*y*, −*z*.

## References

[bb1] Bernstein, J., Davis, R. E., Shimoni, L. & Chang, N.-L. (1995). *Angew. Chem. Int. Ed. Engl.* **34**, 1555–1573.

[bb2] Bruker (2005). *SADABS*. Bruker AXS Inc., Madison, Wisconsin, USA.

[bb3] Bruker (2007). *APEX2* and *SAINT*. Bruker AXS Inc., Madison, Wisconsin, USA.

[bb4] Farrugia, L. J. (2012). *J. Appl. Cryst.* **45**, 849–854.

[bb5] Li, W., Yin, H., Wen, L., Li, K. & Fan, W. (2009). *Acta Cryst.* E**65**, o2579. 10.1107/S1600536809038926 PMC2970331 21578016

[bb6] Odabaşoğlu, M. & Büyükgüngör, O. (2006*a*). *Acta Cryst.* E**62**, o2943–o2944.

[bb7] Odabaşoğlu, M. & Büyükgüngör, O. (2006*b*). *Acta Cryst.* E**62**, o4140–o4141.

[bb8] Odabaşoğlu, M. & Büyükgüngör, O. (2007*a*). *Acta Cryst.* E**63**, o1999–o2001.

[bb9] Odabaşoğlu, M. & Büyükgüngör, O. (2007*b*). *Acta Cryst.* E**63**, o2159–o2161.

[bb10] Sheldrick, G. M. (2008). *Acta Cryst.* A**64**, 112–122. 10.1107/S0108767307043930 18156677

[bb11] Sheldrick, G. M. (2015). *Acta Cryst.* C**71**, 3–8.

[bb12] Spek, A. L. (2009). *Acta Cryst.* D**65**, 148–155. 10.1107/S090744490804362X PMC2631630 19171970

