# Supplementary figures and images for: Crystal structure of 3-(3,4-di­methyl­anilino)-2-benzo­furan-1(3H)-one
Source: Acta Crystallogr E Crystallogr Commun. 2015 May 20;71(Pt 6):o413. doi: 10.1107/S2056989015009299 (PMC4459309; doi:10.1107/S2056989015009299)

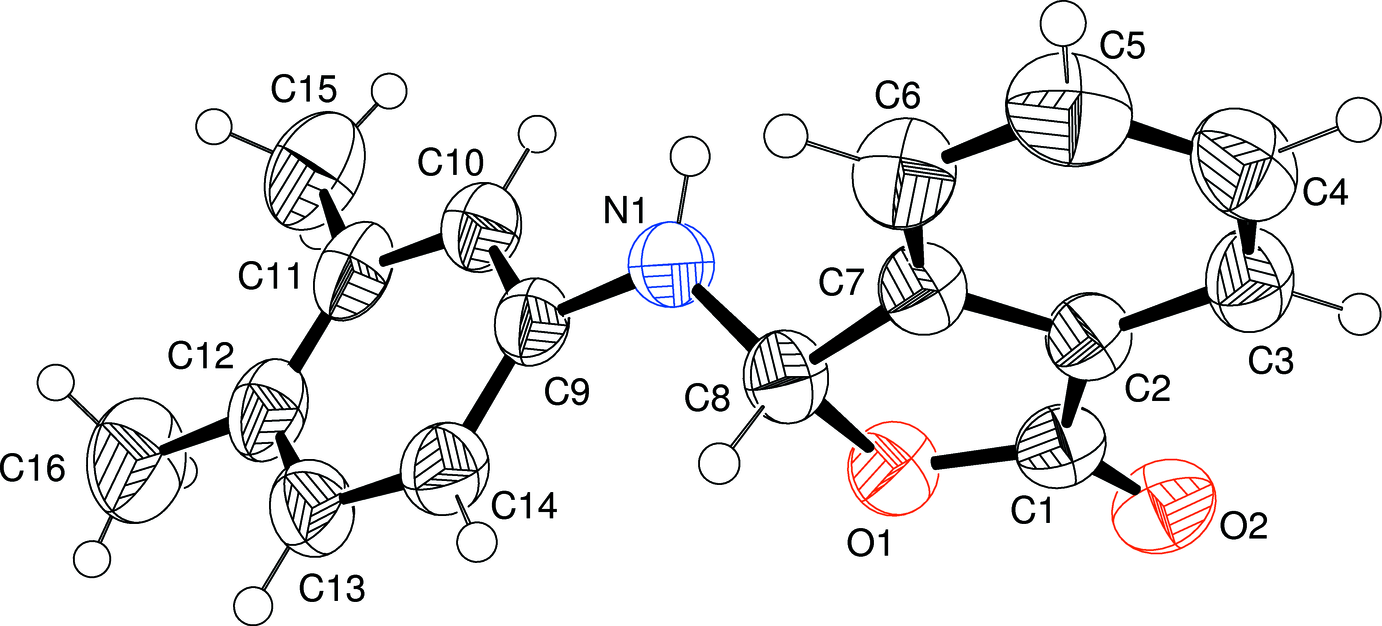

Supplement: Supplementary file 4 [file e-71-0o413-fig1.tif]

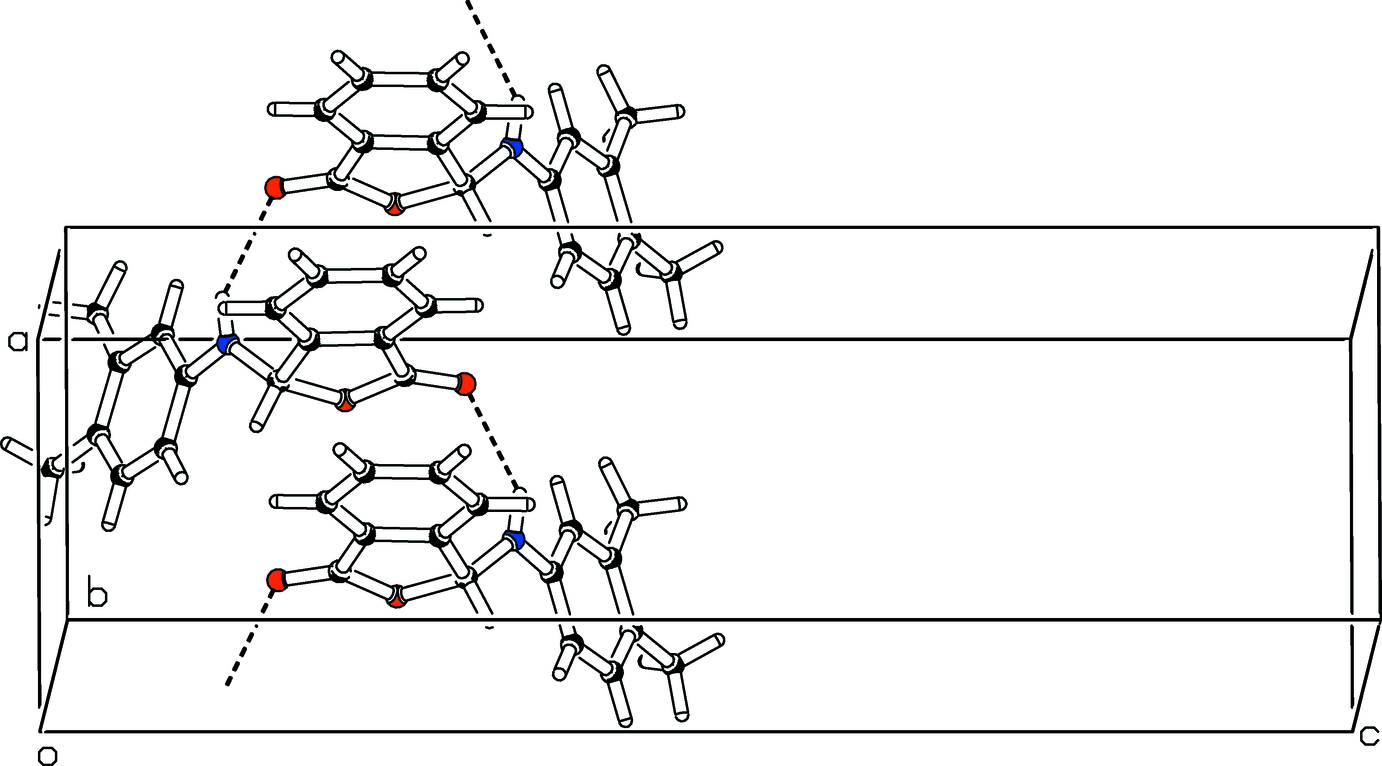

Supplement: Supplementary file 5 [file e-71-0o413-fig2.tif]
